# Patterns of self-medication and intention to seek pharmacist guidance among older adults during the COVID-19 pandemic in Macao: a cross-sectional study

**DOI:** 10.1186/s12889-024-19453-2

**Published:** 2024-07-31

**Authors:** Yu Zheng, Pou Kuan Tang, Hao Hu, Carolina Oi Lam Ung

**Affiliations:** 1 State Key Laboratory of Quality Research in Chinese Medicine, Institute of Chinese Medical Sciences, University of Macau, Macao, China; 2 Department of Public Health and Medicinal Administration, Faculty of Health Sciences, University of Macau, Macao, China; 3 Centre for Pharmaceutical Regulatory Sciences, University of Macau, Macao, China

**Keywords:** Self-medication, Pharmacist, COVID-19, Older adults, Theory of planned behavior, Public health

## Abstract

**Introduction:**

Self-medication was remarkably popular during the COVID-19 pandemic. In older populations, the risk of self-medication is higher. Pharmacists are well positioned to provide public health education and disease prevention. This study aims to explore the self-medication patterns and intention to seek pharmacist guidance among older adults in Macao.

**Methods:**

A face-to-face cross-sectional survey was subsequently performed in March-April 2023 among older adults in Macao. The questionnaire was designed based on the Theory of Planned Behavior (TPB) framework. Multiple logistic regression was used to analyze predictors of self-medication behavior and multiple linear regression analysis to determine whether the TPB construct was the predictor of older adults’ intention to seek guidance from pharmacists.

**Results:**

A total of 412 participants completed the questionnaire. The self-medication rate among older adults in Macao was 64.2%. The most commonly used types of medications were over-the-counter and traditional Chinese medicine, mainly from government anti-pandemic packages. The majority of individuals engaged in self-medication to treat COVID-19 symptoms or prevent COVID-19 infection. The prevalent reasons for self-medication were the perceived non-seriousness of the illness. 85 years old or older and university degree were significantly associated with self-medication behavior. Older adults had moderate intention to seek pharmacist guidance on medication use. The average scores (standard deviation) were 3.43 (1.10) for *Attitude*, 2.69 (0.99) for *Subjective Norm*, 3.56 (1.04) for *Perceived Behavioral Control*, and 3.07 (1.43) for *Intention*. *Attitude*, *Subjective Norm*, and *Perceived Behavioral Control* were all strong predictors of intention, which explained 53% of the variance in intention. In demographic factors, age was identified as a significant predictor of intentions.

**Conclusions:**

Self-medication was widely practiced in Macao during the COVID-19 pandemic. To better control the risks associated with self-medication, the role of pharmacists is paramount. Enhancing the recognition and trust of pharmacists within society, modifying pharmacy management models, and strengthening pharmacists’ self-perception of their profession are all pivotal directions areas to further enhance their role.

**Supplementary Information:**

The online version contains supplementary material available at 10.1186/s12889-024-19453-2.

## Introduction

Self-medication is defined by the World Health Organization (WHO) as treatment of self-recognized disorders or symptoms by use of medicines without prior consultation by a qualified health professional or intermittent/continued use of medicines previously prescribed by a physician for chronic/recurring diseases [1]. Self-medication is a common health-seeking behavior across the globe [2–4]. Individuals resort to self-medication for various reasons which might include prolonged waiting times for medical care, challenges in securing doctor’s appointments, inadequate availability of essential medications, delays in receiving attention, and insufficient capacity in medical facilities, among other associated issues [5]. Furthermore, with the advent of digitalization, people now have convenient access to the internet, allowing them to easily explore their symptoms and find recommendations about possible treatment options [6].

The practice of self-medication has been particularly prominent during the COVID-19 pandemic. A systematic review found the prevalence of self-medication behaviors during the COVID-19 pandemic ranged from 7.14 to 88.3% [7]. Common causes and factors contributing to self-medication practices in the context of COVID-19 included individuals’ fear of contracting the virus and limited access to healthcare services. Self-medication was a cause for concern as it could lead to incorrect medication use, which might exacerbate health conditions, cause adverse reactions or delay necessary medical attention [8]. In particular, during the COVID-19 pandemic when the healthcare facilities were already overburdened, adverse events associated with self-medication might impose further strains on the healthcare system, hindering the ability to respond effectively to the pandemic [9]. Moreover, healthcare misinformation related to the COVID-19 pandemic was spreading at an alarming rate on social media, which could also contribute to unproven or harmful self-medication behavior [10].

Responsible self-medication yields positive outcomes for both the healthcare system and patients [11, 12]. In resource-limited settings, such as during the COVID-19 pandemic, responsible self-medication can alleviate the strain on healthcare systems and minimize the wastage of healthcare resources [13]. Moreover, for patients, it provides a convenient and cost-effective means to prevent and alleviate minor medical conditions while empowering them to take control of their own health management [14]. Nonetheless, it is essential to acknowledge the risks associated with self-medication practices, which include adverse drug reactions, drug interactions, exacerbation of underlying health conditions, and the emergence of antimicrobial resistance [13, 15].

Self-medication among older adults is a significant public health concern, given the likelihood of polypharmacy and their heightened vulnerability to potential drug interactions [16]. Older adults undergo various age-related alterations in their body’s functional systems, which can impact the absorption, distribution, metabolism, and elimination of medications [17]. Impaired hepatic function, diminished renal excretion, reduced body water content, and elevated body fat content contribute to an increased incidence of drug interactions in older adults, rendering them more vulnerable to adverse reactions [18–21]. Moreover, older adults are more likely to have co-morbidities, leading to a higher medication burden and an increased propensity for self-medication practices [21, 22]. Studies have demonstrated that Chinese patients with chronic diseases allocate a greater portion of their healthcare expenditure towards Western and Chinese over-the-counter (OTC) products compared to those without chronic conditions [23]. Similarly, a study conducted in Australia revealed that 80% of patients with chronic diseases relied on OTC products, with a significant proportion misusing these medications in terms of dosage [24]. Extensive utilization of medications and a high prevalence of potentially inappropriate medication use contribute to elevated risks of medication-related problems in older adults during the pandemic due to the proliferation of misinformation on social media and its impact on self-medicating behavior [25].

To support public health with the responsible practice of self-medication, it has been suggested that pharmacists are well-positioned in the community setting to do just that as they are usually the primary source of products for self- medications during the COVID-19 pandemic [7, 26, 27]. This highlights the vital role of pharmacists as essential healthcare professionals in guiding self-medication practices during such crises. Clinicians and nurses are already overwhelmed with their responsibilities, presenting an opportunity for pharmacists to offer collaborative expertise that seamlessly integrates with the existing model of care [28]. Pharmacists play a crucial role in detecting atypical medication usage or irrational prescription patterns within the public [11, 26]. Moreover, community pharmacists have the ability to dispel misinformation about medications and actively promote responsible self-medication practices among the public [29]. However, pharmacists’ extended role in public health in relation to self-medication has remained under-investigated [7].

As the ongoing pandemic continues, self-medication behaviors are expected to exhibit variations across different waves. The heightened risk associated with self-medication among older adults necessitates attention, yet there remains a dearth of pertinent literature. Previous systematic reviews have identified significant variation in outcomes among studies, highlighting the presence of distinct patterns and implications for self-medication in different settings (regions, countries) [7]. On the other hand, although pharmacists can play a proactive role in promoting responsible self-medication, it remains unclear about consumers’ intentions to seek self-medication guidance from pharmacists during COVID-19 and the factors influencing these intentions. Therefore, this study aimed to (1) assess the implementation of self-medication among older adults in Macao in the context of COVID-19; and (2) investigate the intention of older adults to seek pharmacist guidance when self-medicating during COVID-19 and identify the main factors predicting their intention by employing. The overall goal is to promote responsible self-medication and to inform whether community pharmacists play a role in promoting safe and effective medication use in older populations during public health emergencies.

## Methods

A cross-sectional survey was employed to investigate the self-medication behavior and intention for pharmacist-led medication use among older adults in Macao during the COVID-19 pandemic outbreak. The research has been approved by the Panel on Research Ethics of the University of Macau (BSERE19-APP027-ICMS). Following the Strengthening the Reporting of Observational Studies in Epidemiology (STROBE) guideline [30], the reporting of the study is as follows (Supplementary Material 1).

### Study target

Macao, with a population of around 683,100 over 32.9 km^2^, is one of the most densely populated places in the world and a famous tourist destination, exposing the city to a high risk of community transmission and imported cases amid the COVID-19 pandemic. With the change in policy issued on 7th December 2022 regarding measures to prevent and control the pandemic, the number of infections in the city shot up. In response to the fluctuation of the pandemic in Macao and the surrounding areas, the government distributed free “anti-pandemic packages” to all Macao residents since 8th December 2022, which contain antipyretics for self-medication whenever needed [31]. A graded management system for infected individuals was also in place to provide triage for local patients with diverse medical needs through a self-assessment platform [32]. The government made available brief videos of professional medication guidance by Chinese and Western doctors to facilitate the rational purchase and use of painkilling and antipyretic medications. Special populations including the older adults, pregnant women, children and patients with chronic diseases were urged to seek the advice of physicians or community pharmacists before taking such medications [33].

Given the timeline of the pandemic outbreak, the target population of this survey study was people aged 65 years or above who had resided in Macao from December 2022. Considering the population size in Macao, a minimum sample size of 384 respondents would provide a target 5% margin of error for population percentage estimates with a level of 95% confidence.

### Theoretical framework

Currently, there are a lack of existing scales to assess consumers’ intentions and influencing factors regarding seeking self-medication guidance from pharmacists. To facilitate systematic interpretation and analysis of data, as well as to enhance intervention design of pharmacy services, we employed the Theory of Planned Behavior (TPB) as a theoretical framework in our study [34]. The TPB, an extension of the theory of reasoned action, serves as a psychological model elucidating purposive behavior and deliberative decision-making processes [35, 36]. It has been extensively utilized to elucidate and forecast behavioral intentions and health-related behaviors, including the utilization of health services [37, 38]. Within the TPB framework, there are several key constructs, among which behavior (not investigated in this study) and intention serve as the dependent variables, while *Attitude* (A), *Subjective Norm* (SN), and *Perceived Behavioral Control* (PBC) function as the independent variables. Each of these independent variables is underpinned by beliefs. In essence, the TPB posits that an individual’s intention to engage in a behavior is chiefly influenced by their attitude towards the behavior (A), their perception of social norms associated with it (SN), and their perceived ease or difficulty in performing the behavior (PBC) [39].

### Questionnaire design

The design of the questionnaire was developed with reference to (1) the results derived from the systematic review on self-medication behavior during COVID-19 [7], (2) the literature related to pharmacist-directed medication administration [39–43], and (3) studies that used the theory of planned behavior as a theoretical framework [44, 45].

The questionnaire consisted of three main parts. In part A, participants were asked to confirm whether they were residing in Macao since December 2022 and to answer 9 questions regarding their demographic information. These questions were based on factors associated with self-medication behavior obtained from a systematic review [7], including gender, age, marital status, highest level of education achieved, employment status, monthly income, chronic disease status, prescription drug use, and self-reported health status. The classification of these variables was based on related literature and local data from Macao, such as age [46], education level [22, 47], monthly income [47, 48], and the number of prescription drugs and chronic diseases [49, 50].

In section B, participants were informed about self-medication during COVID-19. For this study, self-medication was defined as taking medications to prevent or treat physical symptoms without the guidance of any medical professional. The time frame for self-medication was restricted to after December 2022 according to the time period of the COVID-19 pandemic outbreak. Respondents were first determined whether they had practiced self-medication behavior; if not, the questionnaire proceeded to the section C; if so, they continued to answer relevant questions including purpose of self-medication, type of medication, source of medication, source of information, reasons for self-medication and adverse effect profile. The question options were summarized from the results of the systematic review [7] and added local cultural features of Macao [31]. In each question participants were invited to provide additional feedback through a free-text response box.

**Fig. 1:**
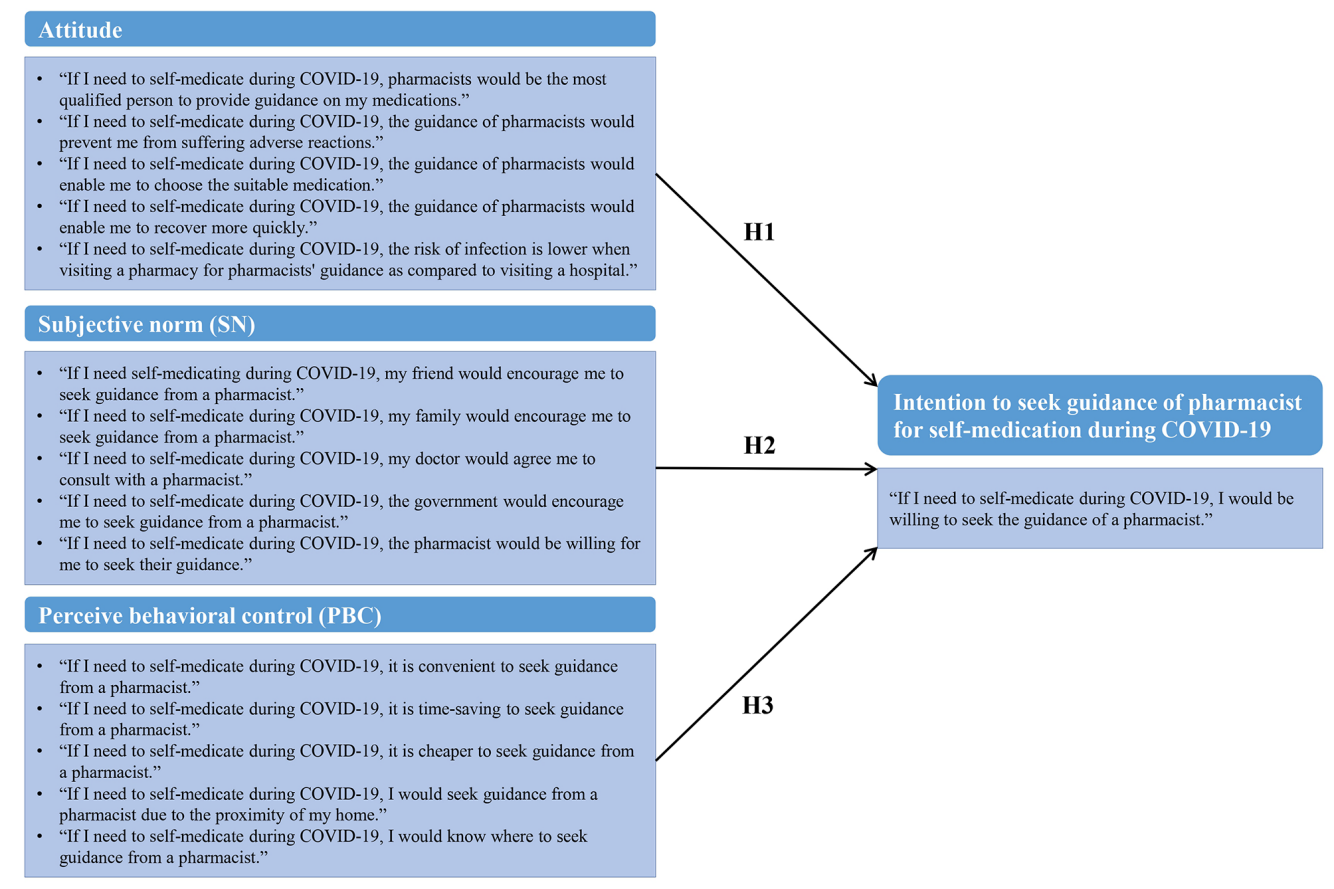
Research model of older adults’ Intention to seek guidance of pharmacist for self-medication during COVD-19

In Section C, the questionnaire was developed using the TPB framework to explore the role of pharmacists in guidance for self-medication during COVID-19. In this study, *Attitude* (A) referred to the degree to which the general public had a favorable or unfavorable evaluation of seeking pharmacist guidance when self-medicating during the pandemic, *Subjective Norm* (SN) referred to the belief about whether peers and those important to them believe they should seek pharmacist guidance when self-medicating during a pandemic, and *Perceived Behavior Control* (PBC) referred to the perception of general public on the level of ease or difficulty of seeking pharmacist guidance when self-medicating during the pandemic. (Fig. 1)

The study hypotheses are, therefore, as follows:

#### H1

Favorable *Attitude* is a positive and significant predictor of the intention to seek pharmacist guidance when self-medicating during COVID-19 pandemic.

#### H2

Positive *Subjective Norm* is a positive and significant predictor of intention to seek pharmacist guidance when self-medicating during COVID-19 pandemic.

#### H3

Strong *Perceived Behavior Control* is a positive and significant predictor of intention to seek pharmacist guidance when self-medicating during COVID-19 pandemic.

In Section C, there were four sub-sections, each of which had a number of statements measuring A (5 statements), SN (5 statements), PBC (5 statements), and *Intention* (1 statement). A 5-point Likert scale with the options strongly disagree, disagree, neutral, agree, and strongly agree was used to gauge the level of agreement among respondents. To prevent partial responses and missing data, all of the questions were designated as obligatory answer items.

### Development of the questionnaire

To reduce sampling bias owing to language barriers, the self-administered structured questionnaire utilized in this study was bilingually created in English and Chinese. In accordance with Leavy’s recommendations, the questionnaire underwent two rounds of pilot studies [51]. The original instrument was evaluated in a focus group by three researchers and three health professionals who were experienced in quantitative studies and public health measures and fluency in both languages. Adjustments were made to the wordings based on their suggestions to improve clarity. The revised instrument was then pilot tested on a convenience sample of 10 individuals in the target age group to further assess content validity, content consistency understanding, flawed questions, and time taken to complete. They all concurred that the questions were clear and simple to comprehend, supporting the face and content validity of the survey. There was no need to change the original statements or add new ones. The pilot test responses were not included in the study data for analysis.

### Data collection

Considering the likelihood of older adults being unfamiliar with electronic questionnaires, face-to-face questionnaires were used for data collection. From 4th March to 10th April 2023, four investigators collected questionnaire data from older adults at different streets, parks, and senior centers using convenience sampling. All investigators received systematic training prior to the survey, including background and purpose of the study, significance of the survey, and content of the study. During the training, each question in the survey questionnaire was explained in detail to the investigators to enable them to interpret the questions in a standardized manner and address any doubts of the participants.

To minimize bias, the questionnaires were conducted in parks and senior centers across different areas of Macao. This helped obtain information from a diverse group of older adults with different educational and economic levels, leading to a more comprehensive and accurate study result. Investigators presented their student ID cards and explained the purpose of the survey to the participants. After obtaining affirmative consent, participants were provided with the Participant Information Statement, which clearly stated the purpose of the study, potential use of information, and measures taken to protect confidentiality.

Participants’ eligibility was first verified based on their age and residence in Macao. Eligible participants were then asked if they could complete the questionnaire independently. For those able to do so, appropriate support was provided to facilitate independent completion. For those with limited literacy or mobility abilities, the questionnaire was personally dictated, and assistance provided to complete it. The estimated completion time for the survey was 10 min.

### Data analysis

The survey responses were analyzed using the Statistical Package for Social Sciences (SPSS) version 26 software for Windows. Only the completed questionnaires were included for data analysis. For respondents’ demographic characteristics, descriptive analysis (frequencies) was conducted and univariate analysis using Pearson chi-square test was employed to compare the differences in the self-medication behavior among subgroups. Multiple logistic regression was employed to identity predictors of self-medication. Similarly, for the ratings given by the respondents’ on TPB constructs and the sub-items, descriptive statistics (frequencies, means, and standard deviations) were conducted and Spearman’s rho was used to test the correlation of intention with the constructs and the sub-items. In the section of demographic, Pearson’s chi-square test was used to analyze and compare the differences among subgroups in seeking pharmacist consultation intention. Multiple linear regression analysis was conducted on the data to identify predictors of intention, with intention as the outcome factor and *Attitude*, *Subjective Norm* and *Perceived Behavioral Control* as the predictor factors. Whenever the *P*-value is found to be smaller than 0.05, the association would be considered statistically significant at a confidence level of 95%.

## Results

A total of 412 participants completed the questionnaire, resulting in a 100% response rate. Of the respondents, 257 were female (62.4%) and 155 were male (37.6%). As shown in Table 1, the majority of respondents belonged to the 65–75 year age group (*n* = 225, 54.6%), were married (*n* = 290, 70.4%), retired (*n* = 390, 94.7%), and had a monthly income of less than 10,000 MOP (*n* = 349, 84.7%). Approximately two-thirds of the respondents (64.1%) had completed primary education or less, and only 53 (12.9%) had purchased medical insurance. Most of them (*n* = 319, 76.4%) had one or more chronic diseases, and 79.1% (*n* = 326) were taking one or more prescription medications. As for their health status, most participants rated it as good (*n* = 172, 41.7%) or moderate (*n* = 176, 42.7%).

**Table 1:** Demographic characteristics of the study participants (*n* = 412)

Variable	Frequency (%)	Self-medication					Intention										
		Yes n (%)	No n (%)	χ²	*P* value		No n (%)		Not sure n (%)		Yes n (%)		χ²		*P* value		
		265 (64.3)	147 (35.7)				199(48.3)		37(9.0)		176(42.7)						
Gender																	
Female	257 (62.4)	174 (67.7)	83 (32.3)	3.408	0.065		127 (49.4)		25 (9.7)		105 (40.9)		1.155		0.561		
Male	155 (37.6)	91 (58.7)	64 (41.3)				72 (46.5)		12 (7.7)		71 (45.8)						
Age (year)																	
65–74	225 (54.6)	153 (68.0)	72 (32.0)	12.702^**^	0.002		99 (44.0)		16 (7.1)		110 (48.9)		18.395**		0.001		
75–84	150 (36.4)	98 (65.3)	52 (34.7)				81 (54.0)		12 (8.0)		57 (38.0)						
85+	37 (9.0)	14 (37.8)	23 (62.2)				19 (51.4)		9 (24.3)		9 (24.3)						
Marital status																	
Married	290 (70.4)	202 (69.7)	88 (30.3)	12.164^**^	0.002		134 (46.2)		24 (8.3)		132 (45.5)		4.648		0.325		
Single	108 (26.2)	56 (51.9)	52 (48.1)				56 (51.9)		11 (10.2)		41 (38.0)						
Prefer not to indicate	14 (3.4)	7 (50.0)	7 (50.0)				9 (64.3)		2 (14.3)		3 (21.4)						
Education level																	
Primary school or below	264 (64.1)	170 (64.4)	94 (35.6)	9.002^*^	0.029		134 (50.8)		26 (9.8)		104 (39.4)		7.350		0.290		
High school	133 (32.3)	90 (67.7)	43 (32.3)				56 (42.1)		11 (8.3)		66 (49.6)						
Bachelor degree	14 (3.4)	4 (28.6)	10 (71.4)				9 (64.3)		0 (0.0)		5 (35.7)						
Prefer not to indicate	1 (0.2)	1 (100)	0 (0.0)				0 (0.0)		0 (0.0)		1 (100.0)						
Employment status																	
Retirement	390 (94.7)	251 (64.4)	139 (35.6)	1.853	0.396		193 (49.5)		36 (9.2)		161 (41.3)		6.598		0.159		
Working	21 (5.1)	14 (66.7)	7 (33.3)				6 (28.6)		1 (4.8)		14 (66.7)						
Prefer not to indicate	1 (0.2)	0 (0.0)	1 (100.0)				0 (0.0)		0 (0.0)		1 (100.0)						
Monthly income (MOP)																	
≦ 9,999	349 (84.7)	224 (64.2)	125 (35.8)	5.151	0.161		174 (49.9)		33 (9.5)		142 (40.7)		6.021		0.421		
10,000 − 19,999	32 (7.8)	25 (78.1)	7 (21.9)				10 (31.3)		2 (6.3)		20 (62.5)						
≧ 20,000	15 (3.6)	7 (46.7)	8 (53.3)				7 (46.7)		1 (6.7)		7 (46.7)						
Prefer not to indicate	16 (3.9)	9 (56.3)	7 (43.8)				8 (50)		1 (6.3)		7 (43.8)						
Medical insurance																	
Yes	53 (12.9)	37 (69.8)	16 (30.2)	0.959	0.619		19 (35.8)		5 (9.4)		29 (54.7)		6.091		0.198		
No	357 (86.7)	227 (63.6)	130 (36.4)				178 (49.9)		32 (9.0)		147 (41.2)						
Prefer not to indicate	2 (0.5)	1 (50.0)	1 (50.0)				2 (100.0)		0 (0.0)		0 (0.0)						
Number of chronic diseases																	
0	93 (22.6)	55 (59.1)	38 (40.9)	6.761	0.343		43 (46.2)		7 (7.5)		43 (46.2)		12.836		0.381		
1	111 (26.9)	71 (64.0)	40 (36.0)				60 (54.1)		9 (8.1)		42 (37.8)						
2	92 (22.3)	63 (68.5)	29 (31.5)				50 (54.3)		10 (10.9)		32 (34.8)						
3	75 (18.2)	49 (65.3)	26 (34.7)				29 (38.7)		9 (12.0)		37 (49.3)						
4	27 (6.6)	15 (55.6)	12 (44.4)				11 (40.7)		1 (3.7)		15 (55.6)						
5	7 (1.7)	5 (71.4)	2 (28.6)				2 (28.6)		0 (0.0)		5 (71.4)						
> 5	7 (1.7)	7 (100.0)	0 (0.0)				4 (57.1)		1 (14.3)		2 (28.6)						
Number of prescription drugs taken																	
0	86 (20.9)	53 (61.6)	33 (38.4)	3.039	0.804		42 (48.8)		6 (7.0)		38 (44.2)		19.493		0.077		
1	84 (20.4)	52 (61.9)	32 (38.1)				50 (59.5)		6 (7.1)		28 (33.3)						
2	63 (15.3)	45 (71.4)	18 (28.6)				34 (54.0)		6 (9.5)		23 (36.5)						
3	69 (16.7)	45 (65.2)	24 (34.8)				28 (40.6)		10 (14.5)		31 (44.9)						
4	40 (9.7)	28 (70.0)	12 (30.0)				11(27.5)		2 (5.0)		27 (67.5)						
5	23 (5.6)	14 (60.9)	9 (39.1)				11 (47.8)		3 (13.0)		9 (39.1)						
> 5	47 (11.4)	28 (59.6)	19 (40.4)				23 (48.9)		4 (8.5)		20 (42.6)						
Self-rated health status																	
Excellent	24 (5.8)	14 (58.3)	10 (41.7)	4.930	0.424		12 (50.0)		2 (8.3)		10 (41.7)		6.006		0.815		
Good	148 (35.9)	91 (61.5)	57 (38.5)				75 (50.7)		15 (10.1)		58 (39.2)						
Middle	176 (42.7)	120 (68.2)	56 (31.8)				81 (46.0)		17 (9.7)		78 (44.3)						
Bad	49 (11.9)	28 (57.1)	21 (42.9)				21 (42.9)		3 (6.1)		25 (51.0)						
Horrible	14 (3.4)	11 (78.6)	3 (21.4)				9 (64.3)		0 (0.0)		5 (35.7)						
Prefer not to indicate	1 (0.2)	1 (100.0)	0 (0.0)				1 (100.0)		0 (0.0)		0 (0.0)						
Self-medication																	
Yes	265 (64.3)						117 (44.2)		25 (9.4)		123 (46.4)		5.194		0.074		
No	147 (35.7)						82 (55.8)		12 (8.2)		53 (36.1)						

^*^p < 0.05; ^**^p < 0.01

### Self-medication practices during COVID-19 pandemic

**Fig. 2:**
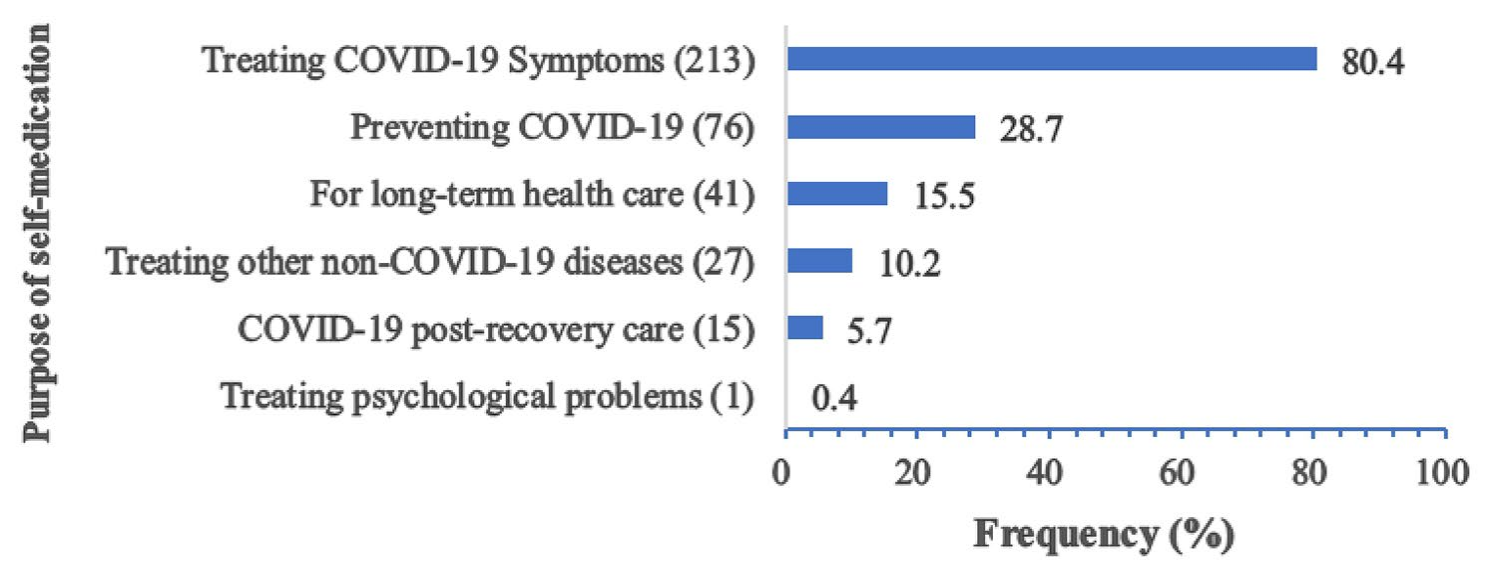
Purpose of self-medication among respondents (*n* = 265)

Overall, 64.3% (*n* = 265) of participants reported self-medicating during the COVID-19 pandemic. As shown in Fig. 2, the majority (80.4%) of those who self-medicated did so to alleviate COVID-19-related symptoms, followed by COVID-19 infection prevention (28.7%). In addition, a small percentage of participants engaged in self-medication during the pandemic to treat non-COVID-19-related illnesses (10.2%) or because they habitually self-administered certain drugs for an extended period (15.5%).

**Fig. 3:**
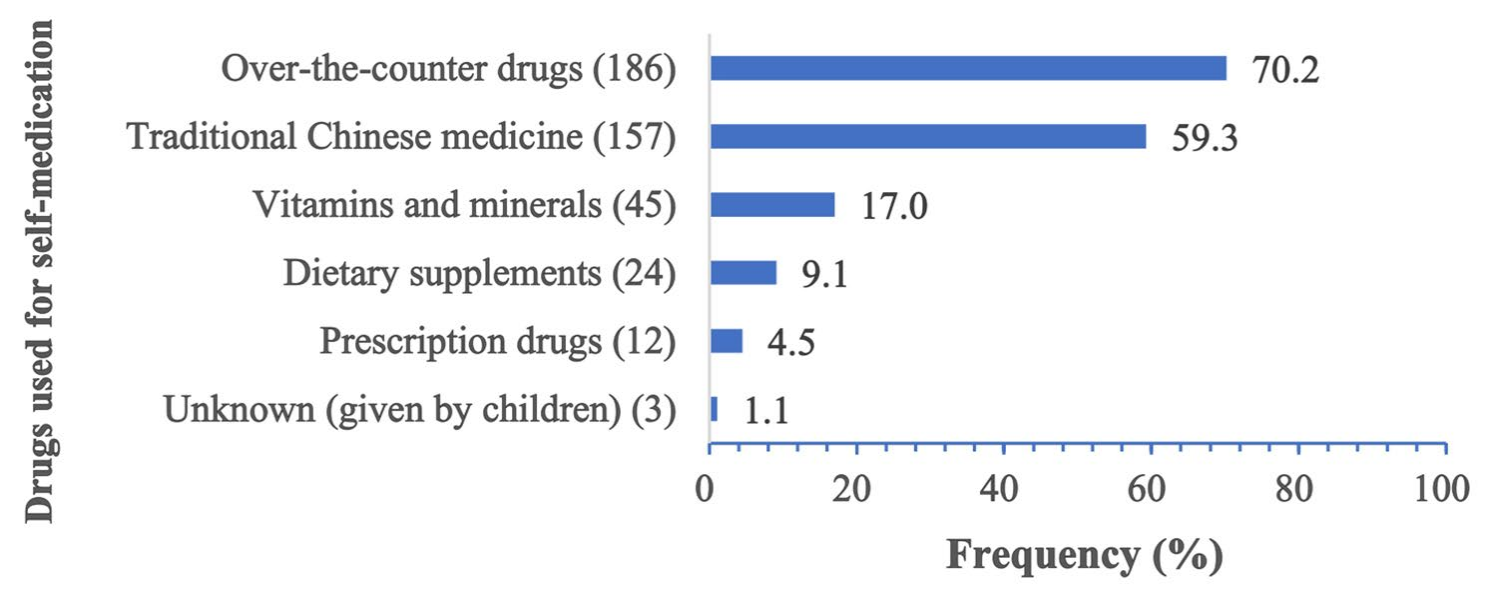
Drugs and supplements used for self‑medication among the respondents (*n* = 265)

The majority of medication types used for self-medication were over-the-counter medications (70.2%) and traditional Chinese medicine (59%). It was followed by vitamins (17%) and dietary supplements (9.1%) (see Fig. 3).

**Fig. 4:**
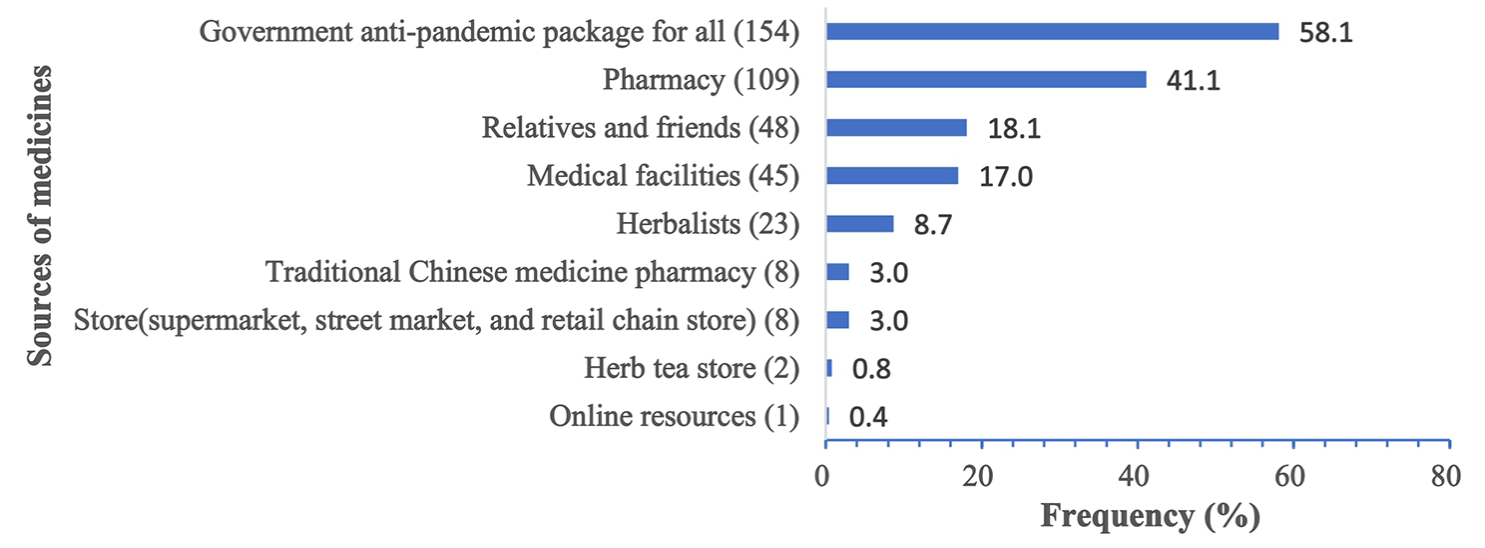
Source of medicines used for self‑medication among the respondents (*n* = 265)

As depicted in Fig. 4, the primary sources of medication for self-medication were government anti-pandemic package (58.1%) and pharmacies (41.1%), according to the majority of respondents. 18.1% of respondents acquired medications from friends or relatives for self-medication, while 17.0% obtained them from medical institutions.

**Fig. 5:**
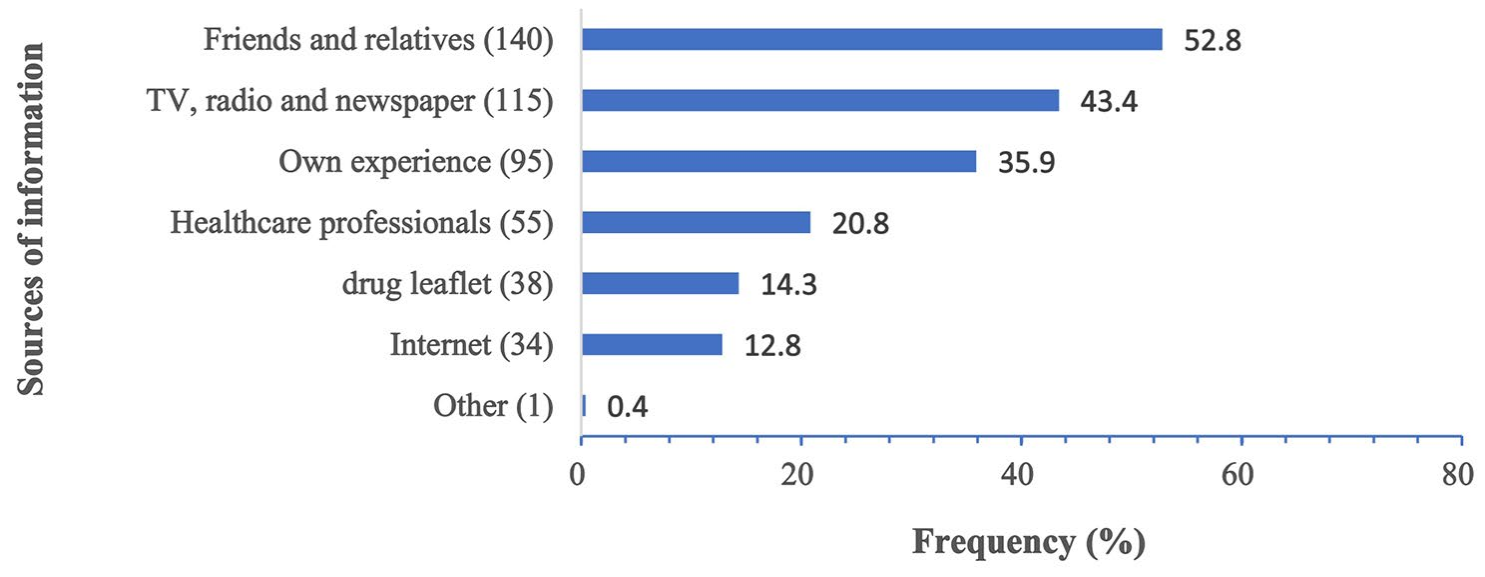
Source of information about self‑medication among the respondents (*n* = 265)

Regarding the source of information for self-medication, the most prevalent sources were friends or family (52.8%) and television or newspapers (43.4%). About one-third of respondents relied on their own experience for taking medication, and healthcare professionals were cited as the source of information by only 20.5% of respondents. Few older adults used the Internet (12.8%) or read drug leaflets (14.3%) to obtain medication information (see Fig. 5).

**Fig. 6:**
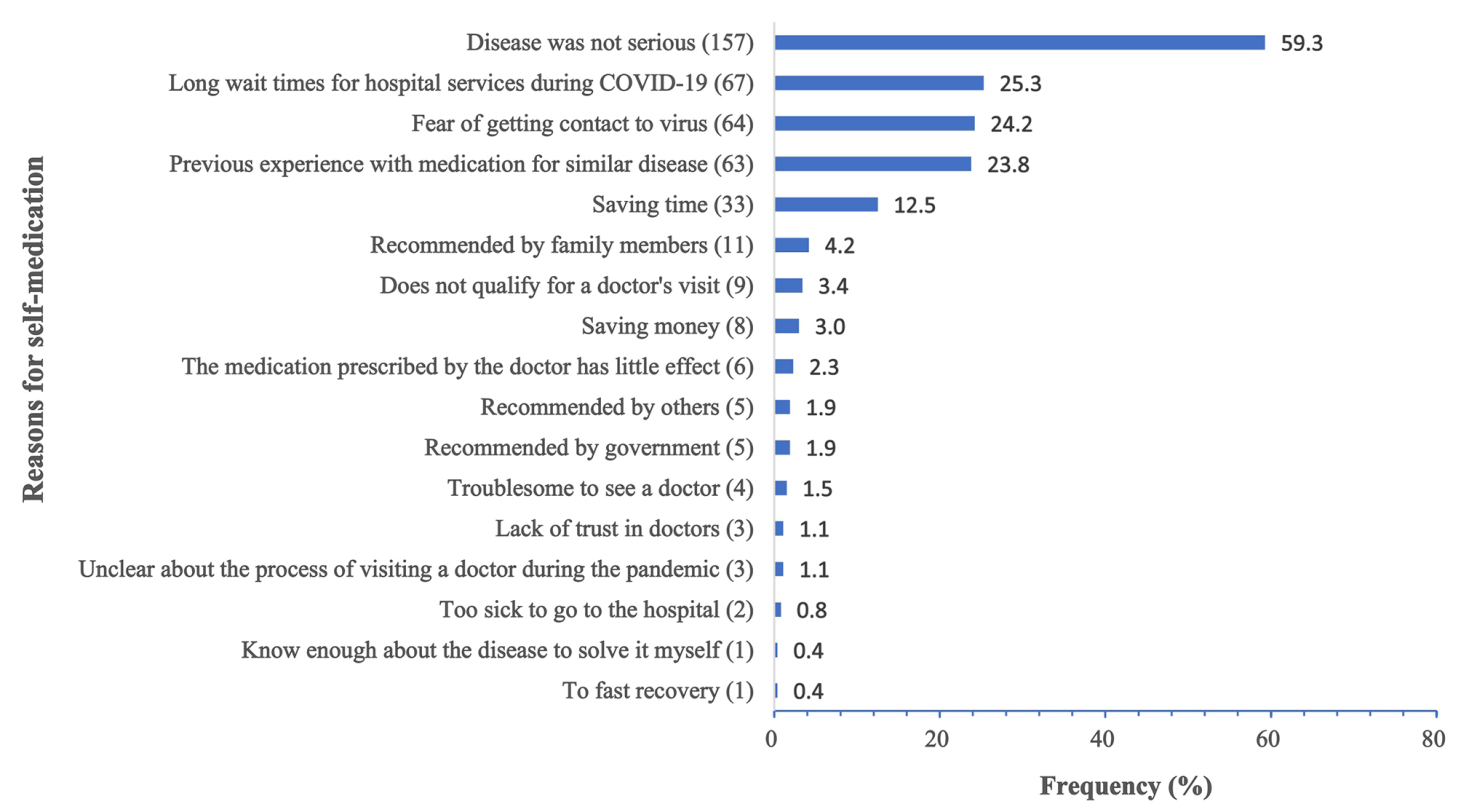
The reasons for the practice of self-medication among the respondents during COVID-19 pandemic (*n* = 265)

Figure 6 displays the list of reasons why the respondents engaged in self-medication. The most prevalent reason for self-medication was the belief that the disease was not severe (59.2%). Similar proportions of respondents self-medicated due to the fear of exposure to the virus (24.2%), long waiting times for hospital services during the COVID-19 pandemic (25.3%), and previous experience with similar medications (23.8%). Other reasons for self-medication included saving time, following advice from others, and not meeting the requirements to schedule a doctor’s appointment.

The adverse reaction rate for self-medication was found to be 14.0% (*n* = 37), with common adverse reactions including sleepiness, dizziness, stomachache, fatigue, nausea, and diarrhea and so on (see Table 2).

**Table 2:** Adverse reactions to self-medication among study participants

Adverse reactions	*N* (%)
Sleepiness	10 (22.7)
Dizziness	10 (22.7)
Not sure	5 (11.4)
Stomachache	3 (6.8)
Fatigue	2 (4.5)
Diarrhea	2 (4.5)
Nausea	2 (4.5)
Other	10 (22.7)
cough, blurred, numbness in the hands, phlegmy, chest tightness, amnesia, back pain, powerlessness, urination, chills	

Univariate analysis showed that age, marital status, and education level were significantly associated with self-medication (*p* < 0.05) (Table 1). Considering the presence of extremely low cell frequencies for certain variables, we performed a sensitivity analysis by excluding them. However, the results remained unchanged despite these adjustments. After correcting for potential confounders by multivariate logistic regression, age above 85 years (*p* = 0.032), and education at bachelor level or higher (*p* = 0.017) were significantly associated with self-medication practices (Table 3). Self-medication was 2.42 times more common among those aged 85 years and older than those aged 65 to 75 years (OR = 2.424; 95% CI: 1.077–5.455). People with a bachelor level education or higher were 4.38 times more likely to self-medicate compared to those with primary education or less (OR = 4.380; 95% CI: 1.309–14.650).

**Table 3:** Predictors of self‑medication among study participants

Variable	aOR	95%CI	*P* value
Age (year)			
65–74	1		
75–84	0.902	0.559–1.455	0.672
85+	2.424	1.077–5.455	0.032
Marital status			
Married	1		
Single	1.635	0.970–2.754	0.065
Prefer not to indicate	2.572	0.853–7.759	0.094
Education level			
Primary school or below	1		
High school	0.943	0.596–1.494	0.804
Bachelor degree or above	4.380	1.309–14.650	0.017
Prefer not to indicate	0.000	0.000	1.000

aOR: Adjusted odds ratio, CI: Confidence interval

### Intention to seek guidance of pharmacist for self-medication

In total, 176 respondents (42.7%) expressed their willingness to seek guidance from a pharmacist when self-medicating during the COVID-19 pandemic. However, 37 respondents (9.0%) were hesitant, and 199 respondents (48.3%) expressed that they did not want to seek guidance from a pharmacist. (Table 1). Among the subgroups, respondents who were male, aged 65–75 years, married, with junior high school education or higher, employed, with a monthly income of over 10,000 MOP (1USD = 8MOP), with health insurance, and who had self-medication during the COVID-19 pandemic had a higher intention to seek guidance from a pharmacist for administering medication. According to the results of the Pearson chi-square test, statistically significant differences were observed in the intention to seek guidance from a pharmacist among age.

### Respondents’ perception about the measurements

The survey responses for each key construct (*Attitude*, *Subjective Norm*, and *Perceived Behavioral Control*) as well as the intention were analyzed, and the percentage of respondents providing positive (strongly agree/agree), neutral, and negative (strongly disagree/disagree) ratings for each statement was presented. Additionally, descriptive statistics including the mean and standard deviation of the ratings for each statement within each TPB key variable and intention were provided in Table 4. The results of Spearman’s rho suggested that each of the items was significantly associated with respondents’ level of intention to seek guidance of pharmacist for self-medication. Moreover, Cronbach’s Alpha analyses were conducted for A, SN, and PBC, yielding values of 0.903, 0.827, and 0.874, respectively. Each of these values exceeded the threshold of 0.7, indicating good internal consistency reliability for each construct.

**Table 4:** Measurement of the TPB constructs

Measures and sub-items of each measure	Mean	S/D	Frequency					Association of the construct subitem with intention		Association of the construct with intention		
			Strongly disagree n (%)	Disagree n (%)	Not sure n (%)	Agree n (%)	Strongly agree n (%)	Spearman’s rho	*P* value	Spearman’s rho	*P* value	
Dependent variable—Intention												
“If I need to self-medicate during COVID-19, I would be willing to seek the guidance of a pharmacist.”	3.07	± 1.43	52 (12.6)	147 (35.7)	37 (9.0)	73 (17.7)	103 (25.0)					
Independent variables—TPB constructs												
Construct 1—Attitude (Cronbach’s alpha 0.903)										0.707	0.000	
“If I need to self-medicate during COVID-19, pharmacists would be the most qualified person to provide guidance on my medications.”	3.25	± 1.36	48 (11.7)	96 (23.3)	72 (17.5)	97 (23.5)	99 (24.0)	0.742	0.000			
“If I need to self-medicate during COVID-19, the guidance of pharmacists would prevent me from suffering adverse reactions.”	3.65	± 1.24	18 (4.4)	71 (17.2)	92 (22.3)	89 (21.6)	142 (34.5)	0.611	0.000			
“If I need to self-medicate during COVID-19, the guidance of pharmacists would enable me to choose the suitable medication.”	3.57	± 1.30	27 (6.6)	78 (18.9)	80 (19.4)	88 (21.4)	139 (33.7)	0.620	0.000			
“If I need to self-medicate during COVID-19, the guidance of pharmacists would enable me to recover more quickly.”	3.41	± 1.26	33 (8.0)	72 (17.5)	107(26.0)	94 (22.8)	106 (25.7)	0.631	0.000			
“If I need to self-medicate during COVID-19, the risk of infection is lower when visiting a pharmacy for pharmacists’ guidance as compared to visiting a hospital.”	3.27	± 1.28	40 (9.7)	80 (19.4)	115 (27.9)	81 (19.7)	96 (23.3)	0.374	0.000			
Construct 2—Subjective norm (Cronbach’s alpha 0.827)										0.517	0.000	
“If I need self-medicating during COVID-19, my friend would encourage me to seek guidance from a pharmacist.”	2.50	± 1.37	115 (27.9)	143 (34.7)	42 (10.2)	57 (13.8)	55 (13.3)	0.441	0.000			
“If I need to self-medicate during COVID-19, my family would encourage me to seek guidance from a pharmacist.”	2.53	± 1.43	119 (28.9)	140 (34.0)	37 (9.0)	48 (11.7)	68 (16.5)	0.457	0.000			
“If I need to self-medicate during COVID-19, my doctor would agree me to consult with a pharmacist.”	2.47	± 1.22	111 (26.9)	107 (26.0)	116 (28.2)	46 (11.2)	32 (7.8)	0.431	0.000			
“If I need to self-medicate during COVID-19, the government would encourage me to seek guidance from a pharmacist.”	2.39	± 1.23	108 (26.2)	153 (37.1)	71 (17.2)	44 (10.7)	36 (8.7)	0.346	0.000			
“If I need to self-medicate during COVID-19, the pharmacist would be willing for me to seek their guidance.”	3.55	± 1.19	30 (7.3)	33 (8.0)	147 (35.7)	83 (20.1)	119 (28.9)	0.346	0.000			
Construct 3—Perceive behavioral control (Cronbach’s alpha 0.874)										0.487	0.000	
“If I need to self-medicate during COVID-19, it is convenient to seek guidance from a pharmacist.”	3.75	± 1.21	14 (3.4)	64 (15.5)	89 (21.6)	89 (21.6)	156 (37.9)	0.427	0.000			
“If I need to self-medicate during COVID-19, it is time-saving to seek guidance from a pharmacist.”	3.71	± 1.25	19 (4.6)	66 (16.0)	86 (20.9)	86 (20.9)	155 (37.6)	0.411	0.000			
“If I need to self-medicate during COVID-19, it is cheaper to seek guidance from a pharmacist.”	3.31	± 1.19	35 (8.5)	54 (13.1)	158 (38.3)	78 (18.9)	87 (21.1)	0.322	0.000			
“If I need to self-medicate during COVID-19, I would seek guidance from a pharmacist due to the proximity of my home.”	3.54	± 1.25	28 (6.8)	72 (17.5)	77 (18.7)	119 (28.9)	116 (28.2)	0.397	0.000			
“If I need to self-medicate during COVID-19, I would know where to seek guidance from a pharmacist.”	3.47	± 1.48	66 (16.0)	61 (14.8)	39 (9.5)	105 (25.5)	141 (34.2)	0.458	0.000			

Overall, of the three key variables of TPB, respondents rated PBC in seeking pharmacist guidance for their self-medication during the pandemic highest (average mean = 3.56 ± 1.04), followed by A (average mean = 3.43 ± 1.01). In contrast, SN (average mean = 2.69 ± 0.99) were rated lower and were negative.

The survey results revealed that for the A construct, respondents generally acknowledged the importance of pharmacist guidance in preventing adverse reactions (mean = 3.65 ± 1.24) and helping them choose suitable medications (mean = 3.57 ± 1.30) when self-medicating during the COVID-19 pandemic. However, they were less inclined to agree that if they needed to self-medicate during the pandemic, pharmacists were the most qualified individuals to provide guidance on their medications (mean = 3.25 ± 1.36) and that visiting a pharmacy for pharmacist guidance carried a lower risk of infection compared to visiting a hospital (mean = 3.27 ± 1.28). Approximately half of the respondents (48.5%) believed that pharmacist guidance when self-medication would contribute to a faster recovery, while 27.9% remained uncertain.

In terms of the construct of *Subjective Norm*, respondents indicated a higher agreement that seeking guidance from pharmacists when self-medicating during the COVID-19 pandemic was what pharmacists expected them to do (mean = 3.55 ± 1.19). However, they were more likely to disagree that their friends (mean = 2.50 ± 1.37), family (mean = 2.53 ± 1.43), and doctors (mean = 2.47 ± 1.22) would expect them to seek guidance from pharmacists when self-medicating during the pandemic. Furthermore, only 19.4% of respondents agreed that the government would expect them to seek guidance from pharmacists when self-medicating during the pandemic.

In terms of *Perceived Behavioral Control*, respondents were more likely to consider it convenient (mean = 3.75 ± 1.21) and time-saving (mean = 3.71 ± 1.25) to seek pharmacists’ guidance while self-medicating during the pandemic. In contrast, respondents expressed lower agreement with the statements that they sought pharmacists’ guidance that was close to home (mean = 3.54 ± 1.25) or that they knew where to find a pharmacist (mean = 3.47 ± 1.48). 40% of respondents agreed that seeking guidance from pharmacists for self-medication would be cheap for them, while a similar proportion (38.3%) expressed uncertainty regarding this matter.

### Results of multiple regression

Hypothesis testing was done by multiple linear regression (Table 5). In Model 1, only the three TPB constructs were included as independent variables. The coefficients demonstrated that A, SN, and PBC were significant predictors of intention (all *P* ≦ 0.001). Therefore, hypotheses H1 (favorable *Attitude* is a positive and significant predictor of the intention to seek pharmacist guidance when self-medicating during COVID-19 pandemic), H2 (positive *Subjective Norm* is a positive and significant predictor of intention to seek pharmacist guidance when self-medicating during COVID-19 pandemic), and H3 (strong *Perceived Behavior Control* is a positive and significant predictor of intention to seek pharmacist guidance when self-medicating during COVID-19 pandemic) were supported. Model 1 explained 53.0% of the variance in intention to seek pharmacist guidance when self-medicating during the pandemic (*R* = 0.728, adjusted R^2^ = 0.530, F = 153.534, d.f.=3, *p* < 0.001). Among the three statistically significant predictors, *Attitude* toward seeking pharmacist guidance on medication had the strongest effect on intentions (β = 0.54, *p* < 0.001) compared to SN (β = 0.16, *p* < 0.001) and PBC (β = 0.14, *p* = 0.001).

**Table 5:** Results of multiple regression analysis

Variables	Unstandardized coefficients		Standardized coefficients beta	t	*P* value
	B	Std. Error			
Model 1 (TPB constructs only)					
(Constant)	-0.64	0.19		-3.38	0.001
TPB constructs					
TPB construct 1 - Attitude	0.71	0.05	0.54	13.01	0.000
TPB construct 2 - Subjective norm	0.23	0.06	0.16	3.58	0.000
TPB construct 3 - Perceived behavioral Control	0.19	0.06	0.14	3.33	0.001
F = 153.534, d.f. = 3, *P* < 0.001, *R* = 0.728, R^2^ = 0.530, adjusted R^2^ = 0.527					
Model 2 (TPB constructs, and control variables)					
(Constant)	-0.29	0.55		-0.52	0.601
Control variables					
Age (year)	-0.05	0.08	-0.02	-0.69	0.488
TPB constructs					
TPB construct 1 - Attitude	0.71	0.05	0.54	13.01	0.000
TPB construct 2 - Subjective norm	0.23	0.06	0.16	3.57	0.000
TPB construct 3 - Perceived behavioral Control	0.19	0.06	0.14	3.21	0.001
F = 115.124, d.f. = 4, *P* < 0.001, *R* = 0.729, R^2^ = 0.531, adjusted R^2^ = 0.526					
Model 3 (Sub-items of the TPB constructs)					
(Constant)	-0.22	0.19		-1.16	0.245
TPB construct 1 - Attitude					
*“If I need to self-medicate during COVID-19*,* pharmacists would be the most qualified person to provide guidance on my medications.”*	0.58	0.06	0.55	10.13	0.000
*“If I need to self-medicate during COVID-19*,* the guidance of pharmacists would prevent me from suffering adverse reactions.”*	0.05	0.08	0.04	0.57	0.570
*“If I need to self-medicate during COVID-19*,* the guidance of pharmacists would enable me to choose the suitable medication.”*	-0.07	0.09	-0.06	-0.76	0.449
*“If I need to self-medicate during COVID-19*,* the guidance of pharmacists would enable me to recover more quickly.”*	0.12	0.07	0.11	1.74	0.083
*“If I need to self-medicate during COVID-19*,* the risk of infection is lower when visiting a pharmacy for pharmacists’ guidance as compared to visiting a hospital.”*	-0.02	0.04	-0.02	-0.57	0.567
TPB construct 2 - Subjective norm					
*“If I need self-medicating during COVID-19*,* my friend would encourage me to seek guidance from a pharmacist.”*	0.08	0.07	0.07	1.07	0.287
*“If I need to self-medicate during COVID-19*,* my family would encourage me to seek guidance from a pharmacist.”*	0.01	0.07	0.01	0.15	0.883
*“If I need to self-medicate during COVID-19*,* my doctor would agree me to consult with a pharmacist.”*	0.09	0.05	0.07	1.73	0.084
*“If I need to self-medicate during COVID-19*,* the government would encourage me to seek guidance from a pharmacist.”*	0.09	0.05	0.08	2.07	0.039
*“If I need to self-medicate during COVID-19*,* the pharmacist would be willing for me to seek their guidance.”*	-0.06	0.05	-0.05	-1.25	0.212
TPB construct 3 - Perceived behavioral Control					
*“If I need to self-medicate during COVID-19*,* it is convenient to seek guidance from a pharmacist.”*	0.11	0.07	0.09	1.55	0.122
*“If I need to self-medicate during COVID-19*,* it is time-saving to seek guidance from a pharmacist.”*	0.01	0.07	0.01	0.09	0.929
*“If I need to self-medicate during COVID-19*,* it is cheaper to seek guidance from a pharmacist.”*	-0.05	0.05	-0.05	-1.16	0.248
*“If I need to self-medicate during COVID-19*,* I would seek guidance from a pharmacist due to the proximity of my home.”*	-0.03	0.06	-0.02	-0.42	0.674
*“If I need to self-medicate during COVID-19*,* I would know where to seek guidance from a pharmacist.”*	0.15	0.04	0.15	3.74	0.000
F = 43.721, d.f. = 15, *P* < 0.001, *R* = 0.790, R^2^ = 0.624, adjusted R^2^ = 0.609					

According to the Pearson chi-square test results, age significantly influences the intention to seek guidance from a pharmacist. Consequently, in Model 2, age is included as a predictor of intention alongside the TPB constructs (A, SN, and PBC). The coefficient showed that A, SN, and PBC were significant predictors of intention (all *p* ≦ 0.001), whereas age was not significant (*p* > 0.05). Model 2 had an R² value of 0.531, indicating that 53.1% of the variance in intention was explained by the independent variables (*R* = 0.729, adjusted R^2^ = 0.531, F = 115.124, d.f.=4, *p* < 0.001). The inclusion of age resulted in an R² change of 0.001, suggesting that it did not significantly enhance the model’s explanatory power.

To further assess the model’s robustness, we conducted sensitivity analyses. By adding other independent variables (e.g. gender, marital status, education level, and monthly income), we observed changes in the effects of the original independent variables on the dependent variable and the impact of the new variables on the model. The Supplementary Material 2 presents the model outcomes after including different variables, revealing no significant findings. This series of robustness tests indicates that the influence of A, SN, and PBC on intention remains unchanged.

To gain a more specific understanding of the impact of each concept on the dependent variable and their unique contributions to the model, all subitems of A, SN, and PBC were calculated as independent variables in Model 3. The coefficients indicated that 1 out of 5 *Attitude* subitems, 1 out of 5 *Subjective Norm* and 1 out of 5 *Perceived Behavior Control* subitems were significant predictors of intention (all *p* < 0.05). Model 3 explained 60.9% (*R* = 0.790, adjusted R^2^ = 0.609) of the variance in intention to seek pharmacist guidance on self-medication during the pandemic. Based on Model 3, the most influential predictors were, in descending order: (1) A - " If I need to self-medicate during COVID-19, pharmacists would be the most qualified person to provide guidance on my medications. " (β = 0.58, *p* < 0.001); (2) PBC - " If I need to self-medicate during COVID-19, I would know where to seek guidance from a pharmacist.” (β = 0.15, *p* < 0.001); and (3) SN - " If I need to self-medicate during COVID-19, the government would encourage me to seek guidance from a pharmacist.” (β = 0.09, *p* < 0.05). (Table 5)

## Discussion

This study found that 64.3% of older adults self-medicated during the COVID-19 pandemic primarily with over-the-counter drugs and traditional Chinese medicine obtained from pharmacies. Predictors of self-medication were the older age (over 85 years) and higher education (bachelor’s degree and above). Regarding seeking pharmacist guidance, older adults showed mixed intentions, with approximately equal percentages expressing positive (42.7%) or negative (48.3%) intentions, and a small proportion (9.0%) remained indecisive. Older adults exhibited generally positive A and PBC toward seeking pharmacist guidance for self-medication during the pandemic, and were less influenced by social pressure. These factors collectively influence older adults’ willingness to seek guidance from pharmacists. The findings might help inform strategies for improving medication use practices among older adults and promoting public health, as well as facilitating pharmacists’ support of public health initiatives during the pandemic.

### Self-medication practices in older adults during the COVID-19 pandemic

In the few studies that investigated self-medication behavior among older adults during the pandemic, the prevalence of self-medication in this study (64.3%) was slightly higher compared to other countries such as Turkey (48.7%) [22] and Iran (56.4%) [52]. Simultaneously, a systematic review of self-medication among older adults conducted in 2014 also showed that most studies reported that the percentage of respondents who self-medicated ranged from 20 to 60% [53]. This variation could stem from cultural disparities between nations, alongside differences in healthcare infrastructure, accessibility of healthcare services, and regulations on medication usage. In some cultures, traditional medicine (such as Traditional Chinese Medicine and herbal remedies) has a long history and widespread acceptance, leading many to choose these methods for self-medication [54]. In regions with underdeveloped healthcare systems, where health insurance coverage is insufficient or medical services are scarce, expensive, or difficult to access, especially during the COVID-19 pandemic, people are more likely to opt for the more affordable and convenient option of self-medication [55]. Additionally, in areas where pharmacies are widespread and medications (including over-the-counter drugs) are easily accessible, this convenience encourages self-medication [7]. In contrast, regions with strict regulations and enforcement on the supply of over-the-counter drugs tend to have lower rates of self-medication [56]. However, it is evident that self-medication among older adults is a serious public health issue, which has been widespread worldwide for a long time and has been significantly prevalent during pandemics with confusing healthcare systems. This underscores the importance of individuals being well-informed to mitigate the risks associated with self-medication.

The most frequent types of medications used for self-medication in this study were over-the-counter medications and traditional Chinese medicine. This is consistent with the findings of the two studies from Turkey and Iran already cited [22, 52], where painkillers were the most popular medications taken by older persons during the pandemic but neither study indicated the use of herbal medicines. On the other hand, studies conducted in Indonesia [57], Mexico [58], and Pakistan [59] revealed that relatively low rates in the use of herbal medicines. However, other research on self-medication during the pandemic found that in addition to over-the-counter medications including analgesics [60–62], antipyretics [61, 63, 64], etc. and herbal medicine [65–68], antibiotics [69–71], vitamins [58, 72–75], and dietary supplements [76–78] were also often used. Attention should be drawn to the fact that, similar to most studies [61, 64, 68, 79], the diversity of drugs used for self-medication in this study implied the potential risk of drug interactions.

Notably, the two principal categories of medications, namely over-the-counter drugs and herbal remedies, may yield interactions or adverse effects with one another, thereby influencing the treatments’ efficacy and possibly yielding unforeseen outcomes [80]. For instance, St. John’s Wort, a herbal supplement often employed for alleviating depression, is known to engender interactions with antidepressant agents such as selective serotonin reuptake inhibitors and monoamine oxidase inhibitors [81]. Such interactions may potentially diminish the potency of antidepressants while concurrently augmenting the risk of serotonin syndrome. The elderly, as a special group with multiple basic illnesses or long-term prescriptions, require more awareness of such drug-related problems.

Self-medication among older adults during the pandemic was significantly associated with age and education level in our study. In terms of age, self-medication behaviors were more likely to occur in older adults aged 85 years or older compared to those aged 65–75 years. Similarly, the older the age, the more likely self-medication was found in other studies [82, 83]. This finding in the present study could be explained that the hassle of seeking medical care due to the physical inconvenience of the older adults with increasing age may lead to a preference for family-guided self-medication [84].

In terms of education level, older adults in this study with a bachelor’s degree were three times more likely to self-medicate than those with a primary school degree, possibly due to their higher level of knowledge about diseases and medications, which leads to a greater inclination towards managing minor health issues independently [85–87]. This finding aligns with studies conducted in the general populations of Togo [72], Hong Kong [65] and Australia [88]. Similarly, Heshmatifar et al. also found that during the pandemic, the level of education also significantly associated with self-medication behavior of older adults [52]. However, there are also studies indicating that individuals with lower education levels are more likely to self-medicate, potentially due to restricted access to medical resources or financial constraints [66, 89, 90]. Overall, these findings indicate that the relationship between education level and self-medication is complex and may vary depending on factors such as the study population and healthcare system disparities.

The finding that information on self-medication among older adults primarily came from their relatives and friends in our study is consistent with a study conducted in Nigeria [66]. This may be attributed to the limited literacy and social exposure of older adults, leading them to rely more on medication guidance from their children. In contrast to the study by Saleem et al. [61], older individuals have limited access to electronic devices, such as cell phones, and thus rely more on traditional sources of information, such as television and newspapers, in addition to receiving information from family members. Therefore, it is necessary for the authorities to consider the major ways of accessing information for older adults when conducting public education campaigns.

The percentage of professionals as a source of self-medication information in the survey was only 20%. During the pandemic, there has been an overwhelming and indiscriminate amount of health information available, while professionals remain the most trusted source of health information [91]. Therefore, there is a need to raise awareness among older adults about seeking guidance from professionals when they intend to self-medicate.

### Older adults’ intentions for pharmacist guidance: predictors and recommendations

The findings reaffirm that pharmacists should be positioned as key players in public health measures to address self-medication behaviors during the pandemic. In comparison to other healthcare professionals, community pharmacists are in a unique position to offer assistance and counsel to the general public. Most medications for self-medication were purchased at pharmacies and transacted by pharmacists or counter assistants, which reflected the accessibility of pharmacists. Remarkably, community pharmacists also command a high level of public confidence in their capacity to furnish guidance pertaining to over-the-counter medications [92]. Pharmacists were the professionals most frequently mentioned in several self-medication studies for giving information or advice on medications [60, 63, 68]. Although pharmacies have a long history of promoting self-care, COVID-19 offers pharmacists greater opportunities than ever to increase their efforts.

Pharmacists possess the capacity to impart fundamental knowledge pertaining to medications, enhance patients’ comprehension of pharmaceutical agents, recommend suitable dosages, and oversee patients’ therapeutic journeys [93]. Particularly relevant within the context of older adults, who often grapple with polypharmacy challenges, pharmacists’ engagement becomes pivotal to guarantee the safety and efficacy of their self-medication practices [94]. Concurrently, the insights dispensed by pharmacists serve as a compass for patients to discern instances necessitating medical intervention, thereby reducing the additional burden on hospital emergency departments due to mild disease such as coughs and flu [95–97].

Nevertheless, considerable barriers persist that hinder pharmacists from maximizing their potential. In this study, we employed the TPB framework to comprehend the intentions of elderly individuals in Macao seeking guidance from pharmacists, aiming to enhance analytical rigor through a structured approach to comprehending and predicting human behavior. Research grounded in established theories often reveals the underlying mechanisms that drive behavior, facilitating more precise prediction, intervention, and the development of targeted interventions. Moreover, the TPB framework demonstrates strong predictive validity in explaining individuals’ behavioral intentions. Two review studies have shown that the TPB framework is a suitable theory for predicting intentions, explaining an average of 39% of the variance in general intentions and 59% of the variance in the routine clinical practice of health care professionals [98, 99].

Overall, the intention to seek pharmacist guidance was moderate, positive attitude toward seeking pharmacist guidance and low perceived barriers to the behavior were found, but SN was found to be negative. Favorable A, positive SN, and strong PBC were found to facilitate older adults’ intention to seek pharmacist guidance. This profile lined up with earlier studies that employed the TPB framework to examine health behaviors [39, 100, 101]. Also, within the sub-item analysis, it was observed that the intentions of older adults were influenced by their agreement with the qualifications of pharmacist guidance and their knowledge of where to seek pharmacist guidance services. These revealed a low level of familiarity and recognition of pharmacists among the older population.

*Attitude* of older adults in Macao toward obtaining advice from a pharmacist when self-medicating during the pandemic was an essential and strongest predictor of their intentions. In earlier pertinent research using TPB model, similar outcomes were discovered [38, 100, 102]. In the sub-item of A, the perception of pharmacists as the most qualified person to provide medication counseling emerged as a predictive determinant of intention. This discovery resonates with observations from a preceding study conducted in Hong Kong. While a majority of people agreed that they would consult a pharmacist before using OTC products, but less than half agreed that pharmacists could play a dominant role in self-care [23].

Building and sustaining favorable attitude regarding pharmacist-directed medication usage should be a priority to increase the intentions to seek guidance from pharmacist when self-medicating in COVID-19 pandemic. However, people’s opinions of pharmacists are still limited to their conventional role of delivering medications and were dubious of pharmacist-patient interactions [103–105]. Consequently, the exploration of strategies aimed at heightening the acquaintance of patients and community residents with pharmacists becomes imperative. Pharmacists can bolster their community presence by actively engaging in community initiatives, offering drug management expertise and health advisory services during health exhibitions, lectures, and similar events. Moreover, pharmacists can forge meaningful connections with patients and community members, delivering tailored drug management guidance and health consultation, thereby solidifying their image as authoritative professionals. Furthermore, the dissemination of pharmacists’ insights, narratives, and professional expertise through social media platforms can effectively capture public interest, underscoring their pivotal role in the realm of healthcare.

The fact that SN was a statistically significant predictor of intention in this study is supported by previous studies [106]. However, the overall score for SN was negative, and few participants felt socially driven to engage in this conduct. Only the sub-item “Pharmacists would expect me to go to them for guidance when I self-medicate during an epidemic” received a favorable score. This phenomenon of pharmacists actively providing guidance was also confirmed in a study conducted in the UK. Pharmacists were enthusiastic about providing alcohol screening programs and providing brief advice to women accessing emergency contraceptives [107]. Poor SN brought by physicians and the government in the intention of older adults to seek pharmacist guidance for self-medication.

Notably, as shown in the regression model 3 above, the government engagement in the SN was a predictor of intention. As a result, interprofessional collaboration should be fostered, where channels and connections between physicians and pharmacists need to be established and nurtured. They can create community-wide collaborative programs which facilitate patients to contact a pharmacist when they are unable to schedule an appointment with a physician [23]. Government, as a significant predictor of intent, should also support pharmacist involvement in primary care and promote their role through patient education, thereby providing individuals with more opportunities to interact and collaborate with pharmacists [23]. Future public health strategies should further leverage the social obligation of pharmacists in public welfare, increase their social recognition, and motivate people to ask for professional guidance, including pharmacists, on appropriate drug use.

PBC was found to be a statistically significant predictor of intention in the current study. This finding was in line with previous studies on pharmacists and patients [38, 100, 102]. The focus should be placed on minimizing their perceived barriers to seeking pharmacist support in order to enhance older people’s intention to do so when they self-medicate. In the sub-item, “I know where to find a pharmacist for medication” was a predictor of intention, however, during the study, it was observed that older adults were not aware of the role of a pharmacist and the availability of pharmacist services in community pharmacies. Therefore, raising the awareness and familiarity of older adults with the professional role of pharmacists is an important consideration in their intention to seek pharmacist guidance.

In the sub-item “It is cheaper to seek pharmacist guidance”, nearly 40% of the participants were unsure while 20% disagreed. This result reflected a lack of clarity or even a misunderstanding about the role of the pharmacist among older adults in Macao. Older adults hold uncertainty about whether to charge for pharmacist-directed medication services. This consideration acknowledges that the existing pharmacy incentive and compensation system primarily emphasizes dispensing rather than providing advice [108]. Pharmacy compensation is typically related to prescription volume, which influences pharmacists’ prioritization of time and resources, as well as the general public’s image of pharmacists. Reimbursement models must be altered in order to facilitate the work of pharmacists and free up pharmacy resources for operations like patient counseling and self-medication guidance.

Barriers to pharmacists’ participation in self-medication direction services during COVID-19 are multifaceted which may also include professional self-perception and inadequate training [26]. From the perspective of pharmacists themselves, the capacity and preparedness of pharmacists to adapt to role changes remain uncertain [109]. Pharmacists should be aware of their social responsibility to support public health during the pandemic. Similarly, pharmacists should be encouraged to participate in ongoing professional development activities, such as continuing education to improve patient interaction services.

### Lessons learnt from Macao experiences

In Macao, the practice of self-medication has evolved in response to government policies and has also been shaped by the traditional Chinese medicine heritage and healthcare system. A multitude of individuals draw upon the efficacy of traditional Chinese medicine as an integral component of their daily healthcare regimen. However, in Macao’s healthcare system, pharmacists possess expertise in drug-related consultations but lack prescription authority. In contrast, the public tends to seek advice from doctors due to their professional status and prescription privileges, which have garnered substantial trust. While the role of pharmacists in self-medication is somewhat restricted, their professional knowledge continues to be indispensable in safeguarding public health. In the future, further enhancing the comprehensive development of the healthcare system and bolstering public trust in pharmacists both stand as pivotal directions for promoting the well-being of Macao residents.

This COVID-19 pandemic could serve as a lesson to reflect on comprehensive approaches to maximize health promotion activities among all healthcare providers, especially in resource-limited settings. For future pandemics, we recommend that pharmacists actively involve their patients in early conversations on the medications they may use for the prevention and treatment of infectious diseases and instruct them appropriately. The findings of this study may assist them in reflecting on and evaluating the burden of self-medication in society and benefit them in developing strategies to curb the problem.

### Future research and limitations

Considering the importance of enhancing responsible self-medication practices through the mentorship of health practitioners, particularly pharmacists, and the research gaps in this area, the findings of this study will drive future contextual and insightful research. Greater research is warranted to explore the value of pharmacists in guiding people to self-medicate as well as how pharmacists can better contribute to public health measures. On the other hand, future research needs to explore environmental factors not considered in this study but that may play a role in predicting the intention of older adults to seek pharmacist guidance when self-medicating during the pandemic within the TPB framework. Qualitative research will complement quantitative research to identify specific reasons for the influence of factors such as A, SN, and PBC.

There are some limitations in survey studies as follows. First of all, the information was only gathered through self-reported surveys, whose results might not be entirely accurate and might be affected by respondents’ social expectations, self-reporting mistakes, and poor recollection. Secondly, it should be noted that this study employed a cross-sectional design, limiting its ability to establish a causal relationship between the key factors and self-medication behavior. Similarly, although the study examined the impact of A, SN, and PBC on intention, the underlying reasons for these influences were not elucidated. Third, the TPB model employed in this study did not consider the prior behavior of older adults in seeking pharmacist guidance for medication use. Incorporating a measure of prior behavior could have provided additional insight into the factors influencing older adults’ intentions and increased the explanatory power of the model.

## Conclusion

Two-thirds of older adults in Macao engaged in self-medication, primarily sourcing their medications from pharmacies. However, the intention of older adults in Macao to seek guidance from pharmacists for self-medication during the pandemic remained at a moderate level. Therefore, the engagement of healthcare administrators and policy makers and the implementation of health education programs that leverage the professional role of pharmacists are essential to regulate and monitor appropriate self-medication practices. In preparation for future pandemics, further research is imperative to investigate the implementation of responsible self-medication practice and unlock the untapped potential of pharmacists in guiding self-medication behaviors.

### Electronic supplementary material

Below is the link to the electronic supplementary material.

Supplementary Material 1

Supplementary Material 2

## Data Availability

The original contributions presented in the study are included in the article/Supplementary Material, further inquiries can be directed to the corresponding author.

## Abbreviations

A: Attitude
OTC: Over-the-counter
PBC: Perceived Behavioral Control
SN: Subjective Norm
SPSS: Statistical Package for Social Sciences
STROBE: Strengthening the Reporting of Observational Studies in Epidemiology
TPB: Theory of Planned Behavior
WHO: World Health Organization
