# Supplementary material for: Patterns of self-medication and intention to seek pharmacist guidance among older adults during the COVID-19 pandemic in Macao: a cross-sectional study
Source: BMC Public Health. 2024 Jul 31;24:2066. doi: 10.1186/s12889-024-19453-2 (PMC11293033; doi:10.1186/s12889-024-19453-2)
Supplement: Supplementary file 2 — Supplementary Material 2 [file 12889_2024_19453_MOESM2_ESM.docx]

| Table 1 Sensitivity analysis about model | | | | | |  |
| --- | --- | --- | --- | --- | --- | --- |
| Variables | Unstandardized coefficients | | Standardized coefficients beta | t | P value |  |
|  | B | Std. Error |  |  |  |  |
| **Sensitivity analysis 1** |  |  |  |  |  |  |
| (Constant) | -0.36 | 0.25 |  | -1.40 | 0.161 |  |
| Gender | -0.17 | 0.10 | -0.06 | -1.72 | 0.087 |  |
| TPB construct 1 - Attitude | 0.71 | 0.05 | 0.55 | 13.15 | 0.000 |  |
| TPB construct 2 - Subjective norm | 0.22 | 0.06 | 0.15 | 3.51 | 0.000 |  |
| TPB construct 3 - Perceived behavioral Control | 0.18 | 0.06 | 0.14 | 3.15 | 0.002 |  |
| F = 116.439, d.f. = 4, P < 0.001, R = 0.731, R^2^ = 0.534, adjusted R^2^ = 0.529 | | | | | | |
| **Sensitivity analysis 2** |  |  |  |  |  |  |
| (Constant) | -0.74 | 0.22 |  | -3.34 | 0.001 |  |
| Education level | 0.07 | 0.09 | 0.03 | 0.85 | 0.398 |  |
| TPB construct 1 - Attitude | 0.71 | 0.05 | 0.54 | 13.00 | 0.000 |  |
| TPB construct 2 - Subjective norm | 0.23 | 0.06 | 0.16 | 3.59 | 0.000 |  |
| TPB construct 3 - Perceived behavioral Control | 0.19 | 0.06 | 0.14 | 3.29 | 0.001 |  |
| F = 115.250, d.f. = 4, P < 0.001, R = 0.729, R^2^ = 0.531, adjusted R^2^ = 0.526 | | | | | | |
| **Sensitivity analysis 3** |  |  |  |  |  |  |
| (Constant) | -0.69 | 0.22 |  | -3.14 | 0.002 |  |
| Monthly income | 0.05 | 0.10 | 0.02 | 0.45 | 0.650 |  |
| TPB construct 1 - Attitude | 0.70 | 0.05 | 0.54 | 13.00 | 0.000 |  |
| TPB construct 2 - Subjective norm | 0.23 | 0.06 | 0.16 | 3.56 | 0.000 |  |
| TPB construct 3 - Perceived behavioral Control | 0.19 | 0.06 | 0.14 | 3.32 | 0.001 |  |
| F = 114.978, d.f. = 4, P < 0.001, R = 0.728, R^2^ = 0.531, adjusted R^2^ = 0.526 | | | | | | |
| **Sensitivity analysis 4** |  |  |  |  |  |  |
| (Constant) | -0.51 | 0.24 |  | -2.13 | 0.034 |  |
| Marital status | -0.09 | 0.09 | -0.03 | -0.98 | 0.329 |  |
| TPB construct 1 - Attitude | 0.71 | 0.05 | 0.54 | 13.03 | 0.000 |  |
| TPB construct 2 - Subjective norm | 0.22 | 0.06 | 0.15 | 3.51 | 0.000 |  |
| TPB construct 3 - Perceived behavioral Control | 0.19 | 0.06 | 0.14 | 3.24 | 0.001 |  |
| F = 115.377, d.f. = 4, P < 0.001, R = 0.729, R^2^ = 0.531, adjusted R^2^ = 0.527 | | | | | | |
| **Sensitivity analysis 5** |  |  |  |  |  |  |
| (Constant) | -0.30 | 0.35 |  | -0.86 | 0.391 |  |
| Employment status | -0.17 | 0.14 | -0.04 | -1.18 | 0.24 |  |
| TPB construct 1 - Attitude | 0.7 | 0.05 | 0.54 | 12.92 | 0.000 |  |
| TPB construct 2 - Subjective norm | 0.23 | 0.06 | 0.16 | 3.63 | 0.000 |  |
| TPB construct 3 - Perceived behavioral Control | 0.19 | 0.06 | 0.14 | 3.25 | 0.001 |  |
| F = 115.606, d.f. = 4, P < 0.001, R = 0.729, R^2^ = 0.532, adjusted R^2^ = 0.527 | | | | | | |
| **Sensitivity analysis 6** |  |  |  |  |  |  |
| (Constant) | -0.48 | 0.36 |  | -1.35 | 0.179 |  |
| Medical insurance | -0.08 | 0.14 | -0.02 | -0.54 | 0.588 |  |
| TPB construct 1 - Attitude | 0.71 | 0.05 | 0.54 | 13.01 | 0.000 |  |
| TPB construct 2 - Subjective norm | 0.22 | 0.06 | 0.15 | 3.44 | 0.001 |  |
| TPB construct 3 - Perceived behavioral Control | 0.19 | 0.06 | 0.14 | 3.31 | 0.001 |  |
| F = 115.025, d.f. = 4, P < 0.001, R = 0.728, R^2^ = 0.531, adjusted R^2^ = 0.526 | | | | | | |
| **Sensitivity analysis 7** |  |  |  |  |  |  |
| (Constant) | -0.76 | 0.21 |  | -3.63 | 0.000 |  |
| Number of prescription drugs taken | 0.03 | 0.03 | 0.05 | 1.33 | 0.183 |  |
| TPB construct 1 - Attitude | 0.70 | 0.05 | 0.54 | 12.83 | 0.000 |  |
| TPB construct 2 - Subjective norm | 0.23 | 0.06 | 0.16 | 3.62 | 0.000 |  |
| TPB construct 3 - Perceived behavioral Control | 0.20 | 0.06 | 0.15 | 3.41 | 0.001 |  |
| F = 115.815, d.f. = 4, P < 0.001, R = 0.730, R^2^ = 0.532, adjusted R^2^ = 0.528 | | | | | | |
| **Sensitivity analysis 8** |  |  |  |  |  |  |
| (Constant) | -0.69 | 0.21 |  | -3.28 | 0.001 |  |
| Number of chronic diseases | 0.02 | 0.04 | 0.02 | 0.52 | 0.605 |  |
| TPB construct 1 - Attitude | 0.70 | 0.06 | 0.54 | 12.84 | 0.000 |  |
| TPB construct 2 - Subjective norm | 0.23 | 0.06 | 0.16 | 3.60 | 0.000 |  |
| TPB construct 3 - Perceived behavioral Control | 0.20 | 0.06 | 0.14 | 3.34 | 0.001 |  |
| F = 115.011, d.f. = 4, P < 0.001, R = 0.72, R^2^ = 0.531, adjusted R^2^ = 0.526 | | | | | | |
| **Sensitivity analysis 9** |  |  |  |  |  |  |
| (Constant) | -0.67 | 0.24 |  | -2.75 | 0.006 |  |
| Self-rated health status | 0.01 | 0.06 | 0.01 | 0.16 | 0.870 |  |
| TPB construct 1 - Attitude | 0.70 | 0.05 | 0.54 | 12.96 | 0.000 |  |
| TPB construct 2 - Subjective norm | 0.23 | 0.06 | 0.16 | 3.57 | 0.000 |  |
| TPB construct 3 - Perceived behavioral Control | 0.20 | 0.06 | 0.14 | 3.33 | 0.001 |  |
| F = 114.883, d.f. = 4, P < 0.001, R = 0.728, R^2^ = 0.530, adjusted R^2^ = 0.526 | | | | | | |
| **Sensitivity analysis 10** |  |  |  |  |  |  |
| (Constant) | -0.59 | 0.26 |  | -2.31 | 0.022 |  |
| Self-medication | -0.03 | 0.10 | -0.01 | -0.30 | 0.764 |  |
| TPB construct 1 - Attitude | 0.70 | 0.05 | 0.54 | 12.92 | 0.000 |  |
| TPB construct 2 - Subjective norm | 0.23 | 0.06 | 0.16 | 3.58 | 0.000 |  |
| TPB construct 3 - Perceived behavioral Control | 0.19 | 0.06 | 0.15 | 3.27 | 0.001 |  |
| F = 114.916, d.f. = 4, P < 0.001, R = 0.728, R^2^ = 0.530, adjusted R^2^ = 0.526 | | | | | | |
